# Development and clinical implementation of eclipse scripting‐based automated patient‐specific collision avoidance software

**DOI:** 10.1002/acm2.12673

**Published:** 2019-07-07

**Authors:** Thomas D. Mann, Nicolas P. Ploquin, William R. Gill, Kundan S. Thind

**Affiliations:** ^1^ Department of Physics and Astronomy University of Calgary Calgary AB Canada; ^2^ Department of Medical Physics Tom Baker Cancer Center Calgary AB Canada; ^3^ Department of Radiation Oncology University of Calgary Calgary AB Canada

**Keywords:** collision detection, eclipse scripting, noncoplanar radiotherapy, patient safety

## Abstract

**Purpose:**

Increased use of Linac‐based stereotactic radiosurgery (SRS), which requires highly noncoplanar gantry trajectories, necessitates the development of efficient and accurate methods of collision detection during the treatment planning process. This work outlines the development and clinical implementation of a patient‐specific computed tomography (CT) contour‐based solution that utilizes Eclipse Scripting to ensure maximum integration with clinical workflow.

**Methods:**

The collision detection application uses triangle mesh structures of the gantry and couch, in addition to the body contour of the patient taken during CT simulation, to virtually simulate patient treatments. Collision detection is performed using Binary Tree Hierarchy detection methods. Algorithm accuracy was first validated for simple cuboidal geometry using a calibration phantom and then extended to an anthropomorphic phantom simulation by comparing the measured minimum distance between structures to the predicted minimum distance for all allowable orientations. The collision space was tested at couch angles every 15° from 90 to 270 with the gantry incremented by 5° through the maximum trajectory. Receiver operating characteristic curve analysis was used to assess algorithm sensitivity and accuracy for predicting collision events. Following extensive validation, the application was implemented clinically for all SRS patients.

**Results:**

The application was able to predict minimum distances between structures to within 3 cm. A safety margin of 1.5 cm was sufficient to achieve 100% sensitivity for all test cases. Accuracy obtained was 94.2% with the 5 cm clinical safety margin with 100% true positive collision detection. A total of 88 noncoplanar SRS patients have been currently tested using the application with one collision detected and no undetected collisions occurring. The average time for collision testing per patient was 2 min 58 s.

**Conclusions:**

A collision detection application utilizing patient CT contours was developed and successfully clinically implemented. This application allows collisions to be detected early during the planning process, avoiding patient delays and unnecessary resource utilization if detected during delivery.

## 1 INTRODUCTION

Potential collisions in radiation therapy are a burden on clinical resources when detected late in the treatment process and, when undetected, a detriment to patient safety and equipment maintenance. Collisions can be difficult to detect during the treatment planning stage, especially with techniques that utilize noncoplanar arcs such as Stereotactic Radiosurgery (SRS) and stereotactic body radiation therapy (SBRT). As the number and complexity of these treatments increase, the probability of a gantry to patient and/or couch collision likewise increases. The purpose of this work is the development and clinical implementation of a simple and automated patient‐specific collision detection application that can be easily incorporated into current clinical workflow.

A common method of collision prevention involves the use of pretreatment simulation on a Linac. This simulation increases total patient time on the Linac, which negatively impacts patient quality of life as well as increases resource utilization for the treatment unit. A potential collision discovered at the time of treatment would lead to the treatment plan being re‐planned and re‐approved, requiring additional staff hours from the Radiation Oncologist, Dosimetrist, and Physicist. The treatment itself will also have to be delayed which is an inconvenience for the patient and could be detrimental to the whole patient treatment outcome. Any additional time that the patient is required to be on the treatment couch also increases patient discomfort due to the rigid nature of immobilization systems. Current strategies to prevent re‐planning make use of gantry and couch angles taken from look‐up tables and predetermined noncoplanar arc arrangements based on the isocenter location in the brain. These arrangements tend to be highly conservative to limit the chance of a collision. Conservative gantry trajectories limit the number of available control points for the dose optimization process which could negatively impact plan quality.

Several solutions have been proposed and developed for collision prediction and avoidance.^1^, ^2^, ^3^ One class of solutions uses simple geometric shape modeling to provide a visual or mathematical approximation of the collision‐free space.^4^, ^5^ The geometric class solution suffers from a lack of patient‐specific modeling and therefore has limited accuracy. Two dominant methods have emerged for incorporating patient‐specific body and plan details into collision prediction models. Surface models of patients are acquired either by three‐dimensional (3D) optical scanning or from patient body contours created during planning computed tomography (CT) simulation. Optical scanning accuracy is limited by the number of cameras, camera positioning, fidelity of the scanning system, length of scan, and room registration technique. Yu et al.^6^ assert a maximum 1.5‐cm measurement error on the body extremities for a 1.8 m tall phantom using a handheld scanner. Cardan et al. report a maximum spatial accuracy of 3 cm for certain components of a 3D scan using a static 3 camera system^7^. CT plan data accuracy is limited by the contouring accuracy, size of the scan, reproducibility, and slice width. Varian Medical Systems (Palo Alto, CA, USA) has provided their own solution in the form of HyperArc™, which allows the user to select from a set of known possible trajectories for a predefined immobilization system. Use of HyperArc™, however, is limited to the Encompass™ immobilization system from Qfix (Avondale, Pennsylvania, USA). Aside from the vendor solution provided by Varian, other solutions to collision avoidance suffer from a lack of integration with the clinical treatment planning system and clinical workflow.

In this work, we showcase easy‐to‐use and effective integration of a collision avoidance application with current clinical workflow. This application was created using eclipse scripting application programming interface (ESAPI) (Varian Medical Systems). ESAPI is highly integrated with the Eclipse treatment planning system and provides access to patient‐specific plan parameters. Polygon mesh geometry from the patient body and couch contour is used to perform the collision check with an in‐house collision detection algorithm described in the methods. The application acts as a stand‐alone tool accessible from the eclipse treatment planning system (TPS), which allows for simultaneous use of this application and the Eclipse TPS. Furthermore, this is the first collision detection application to utilize automatic registration of a variety of immobilization devices to patient anatomy, for both visual and numerical verification, all contained within the treatment planning system.

## 2 MATERIALS AND METHODS

### 2.1 Collision avoidance application

#### 2.1.1 Varian edge radiosurgery system CAD model

**Figure 1:**
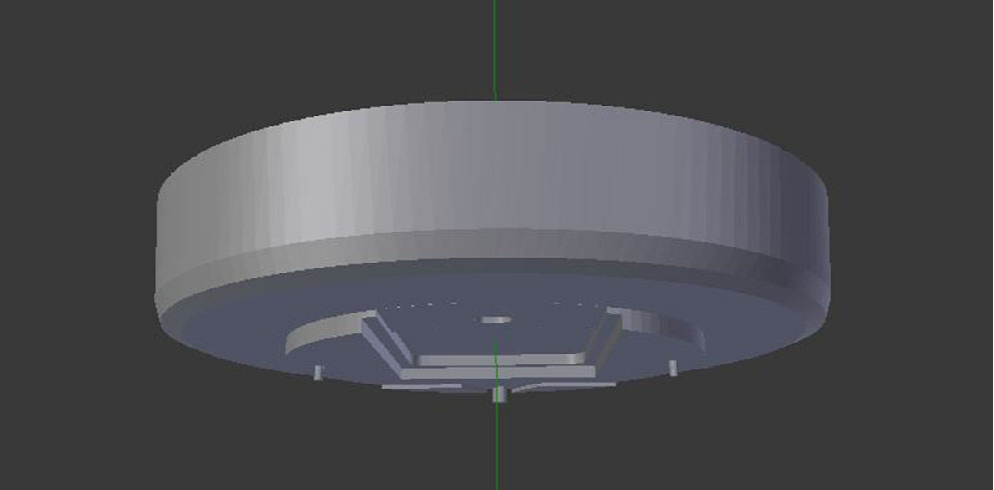
Computer aided design model of a Truebeam Linac head created in SolidWorks.

The collision avoidance application uses a complex model of the gantry head composed of 1639 triangle vertices. This computer‐aided design (CAD) model of the gantry head was created in SolidWorks (Dassault Systèmes, Vélizy‐Villacoublay, France) using physical measurements of the gantry (Fig. 1). For the outer plastic casing, measurements were taken of the head circumference at various heights and linearly interpolated to determine the circumference between these measured points. Fourteen centimeter of the gantry head casing, most relevant to collision prevention, was modeled. Limited gantry modeling allows for a decrease in calculation time. For the gantry face, all major projections, including the locking pins and horseshoe tray insert, were measured for their diameter and projection distance as seen in Fig. 1.

#### 2.1.2 Patient‐specific treatment plan parameters

**Figure 2:**
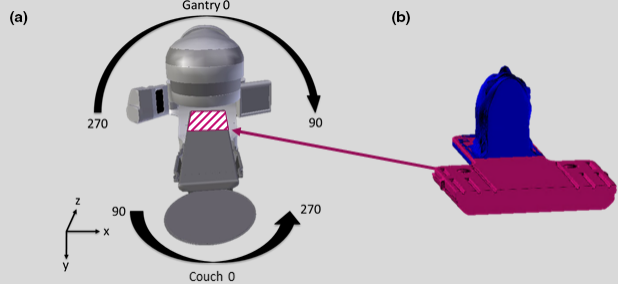
(a) Varian International Electrotechnical Commission gantry and couch coordinate system used, shown with the fixed digital imaging and communications in medicine patient coordinate system. (b) Computed tomography body contour containing the head of the Orfit board with the registered additional couch structure.

Patient plan information to be used in the collision avoidance check is retrieved automatically from the treatment planning system using Eclipse Scripting. Data are accessed by patient ID number, course, and plan. Once a plan is chosen, the associated body and couch contours, plan isocenter, and treatment beams are extracted from the Eclipse treatment database. Contours are saved as geometric mesh structures composed of triangle vertices and indices defining face orientations. The algorithm uses a fixed orthogonal digital imaging and communications in medicine patient‐based coordinate system with the isocenter as the origin and the patient body as the rigid frame of reference. All translations and rotations are applied to the gantry model. Gantry and couch rotations are in standard Varian International Electrotechnical Commission coordinates as shown in Fig. 2(a).

#### 2.1.3 Collision detection algorithm

Each structure used by the algorithm is segmented using oriented bounding boxes (OBBs) into a binary tree hierarchy (BTH) using top‐down segmentation methods. BTH tree traversal collision detection methods are then used to find the minimum distance between structures. This type of algorithm improves efficiency over a simple point‐to‐point test, especially when dealing with large mesh structures. The time for OBB Tree algorithm completion is given by the following equation^8^:

$$T=N_{v}\times T_{v}+N_{p}\times T_{p}\left(1\right)$$

T: total time for collision detection,

$N_{v}$: number of bounding volume overlap tests,

$T_{v}$: time for testing a pair of bounding volumes for overlap,

$N_{p}$: number of primitive pairs (vertices) tested for collision,

$T_{p}$: time for testing a pair of primitives for collision.

As opposed to a point‐to‐point algorithm, which has an efficiency given by:

$$T={\left(N\right)}^{2}\times T_{p}\left(2\right)$$

N: total number of primitives (vertices) for all structures used.

The use of tree structures minimizes $N_{v}$ and $N_{p}$, the number of bounding volume overlap tests and primitives used for collision detection, especially for non‐colliding orientations. For example, testing a single 360° non‐colliding arc using point‐to‐point took 1:31:31.98 compared to only 00:02.77 for the BTH method. After a beam has been selected for testing, the Linac model OBB is first translated to be offset from isocenter at gantry zero degrees, then the collimator rotation for that beam is applied. Starting from the first control point, the gantry rotation is applied in 5° intervals. Couch rotations are applied to the Linac OBB, keeping the patient body OBB constant throughout the test. The separating axis theorem is used to test if a collision occurs between the Linac and body or couch OBBs. If no collision occurs, the Linac OBB continues along the gantry trajectory until the final control point is reached. If the OBBs intersect, then the algorithm continues through the Tree Hierarchy for each structure to find the two closest vertex points between structures. These points are used to calculate the minimum distance between the structures. A patient safety margin of 5 cm is used to increase the collision detection sensitivity. Any structure within the safety margin of either the couch or the body contour will register the event as a collision and warn the user that the plan is unsafe to deliver.

#### 2.1.4 Automated registration algorithm

The scan length for SRS simulation protocol is commonly limited to the top of head to the bottom of the chin. The majority of collisions between the gantry and patient for SRS will occur on the shoulders or chest region, which means additional information is required for the collision check. In cases where the planning CT does not include an upper body structure besides the head, various sized anthropomorphic body phantoms can be automatically registered to the patient body contour and manually adjusted if needed. Detailed pre‐contoured couch structures can also be automatically registered to the body contour in place of clinical couch structures which might lack detail and accuracy. The automatic registration uses maximum positions in the anterior‐posterior, left‐right, and superior direction of the body contour to align the upper body structure. Typically, the head of the Orfit board is contained within the body contour, an example of which can be seen in Fig. 2(b), which allows for quick registration of an additional couch structure containing relevant portions of the Orfit board and couch bottom.

### 2.2 Graphical user interface

**Figure 3:**
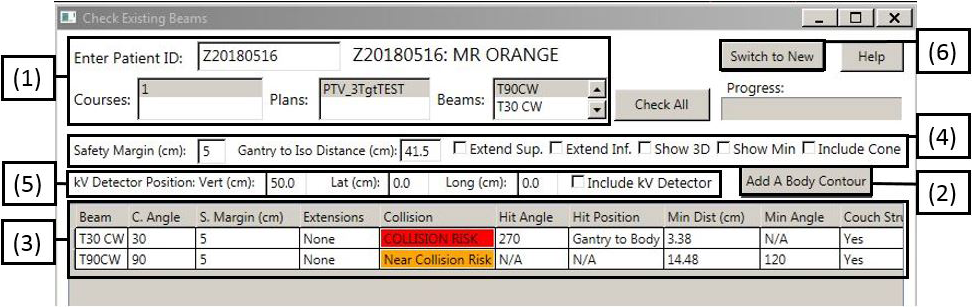
Graphical user interface for the collision detection tool. (1) Patient plan info accessed by ID. (2) Addition of body and couch contours. (3) Results displayed in a data table. (4) Option to modify default parameters, extend contours, or show a three‐dimensional visual display. (5) Ability to add the on‐board imaging system to the collision check. (6) Test gantry limits for a new beam.

**Figure 4:**
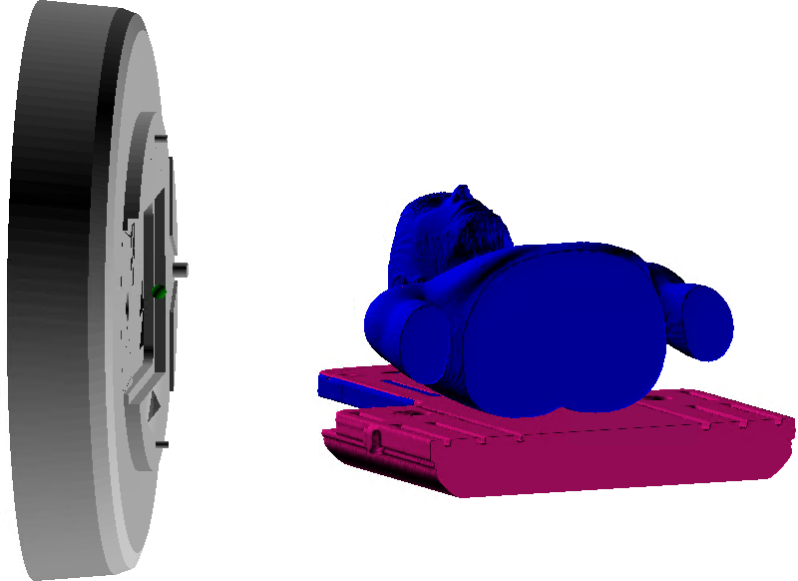
Example of the three‐dimensional visual display option. Display can be rotated to view possible colliding orientations from various angles.

The collision detection application runs as a stand‐alone Eclipse Scripting plug‐in, accessible from the treatment planning system. Patient data are accessed by ID number, treatment course, and plan with an example shown in Fig. 3. There is an option to automatically register detailed couch structures or a body addition if the patient‐contoured structures are not sufficient. These additional structures can be adjusted in a visual window by the user if the registration is unsatisfactory. Users have the choice of testing one or all beams. After the test has been completed, the minimum distance, couch angle, and gantry angle at which it occurred are shown in a data table. Beams are labeled as a “Collision Risk” if the predicted minimum distance is smaller than the chosen safety margin. Beams determined to have a minimum distance within 10 cm of the safety margin are labeled a “Near Collision Risk.” Any beam can also be viewed visually in a separate window by selecting the “Show 3D” or “Show Min” option. An example of “Show Min” is shown in Fig. 4. Plan safety can be determined using visual or numerical feedback from the application. Default parameters such as the safety margin and distance from isocenter to the closest gantry face can be adjusted if necessary. Additional features, (a) variable safety margins, (b) body and couch structure extensions, and (c) On‐Board Imager collision simulation have been developed and incorporated to ensure adequate modeling and collision prevention for a variety of clinical scenarios.

In addition to testing previously created plans for collisions, the user may choose to test the maximum bounds of gantry motion for a specific isocenter and couch angle [Fig. 3(6)]. This allows for inclusion of extra control points during the dose optimization process if the maximum bounds are larger than the clinical plan originally utilized.

### 2.3 Algorithm sensitivity

Gantry and couch model accuracy was assessed using a cuboid calibration phantom. The calibration phantom consisted of solid water placed on top of an Orfit All‐In‐One immobilization board (Orfit Industries, Wijnegem, Belgium). As an end‐to‐end test of model accuracy, the calibration phantom was subjected to clinical SRS patient treatment workflow. The calibration phantom and Orfit board were simulated on a Philips CT simulator and contoured in the Eclipse treatment planning system. A laterally symmetric isocenter was chosen to test for systematic offsets in gantry and phantom positioning. Treatment couch angles were tested in 15° intervals ranging from 90 to 270. At each couch angle, the gantry was incremented by 5° through the maximum possible trajectory for a total of 702 couch and gantry angle combinations. The minimum distance between structures at each possible angle was measured using spring calipers to within millimeter accuracy and compared to the predicted distance. Collision prediction accuracy was also assessed for various safety margins using receiver operating characteristic (ROC) curve analysis as recommended in Cardan et al.^7^ Definitions for the ROC categories are shown in Table 1.

**Table 1:** Categories for receiver operating characteristic analysis with collision detection.

	Calculated collision	Physical collision
True positive (TP)	Yes	Yes
True negative (TN)	No	No
False positive (FP)	Yes	No
False negative (FN)	No	Yes

### 2.4 Collision testing using an anthropomorphic upper body phantom

To simulate a clinical SRS patient setup, an anthropomorphic upper body phantom was immobilized using the current clinical SRS protocol (Orfit Thermoplastic cranial open‐faced mask with the All‐In‐One Baseplate). A planning CT of the phantom's upper body and head was acquired. Four isocenter locations, representing limiting cases in SRS, were tested for gantry clearance at 13 different couch angles ranging from 90 to 270 in 15° intervals. The isocenters consisted of anterior/posterior and left/right combinations located inferiorly at the base of skull to represent a worst‐case collision risk scenario. Algorithm collision prediction accuracy was assessed using ROC curve analysis for each patient test case.

### 2.5 Clinical implementation

After sufficient validation of the algorithm accuracy, it was implemented clinically for all Linac‐based SRS patients as a pretreatment planning stage collision check. The application was accessed from the External Beam Planning tools menu in the treatment planning software to run an automated collision check for each arc associated with the plan. Clinical SRS plans were generally composed of 3–5 noncoplanar arcs, with varying gantry trajectory and couch angles based on the size, location, and number of lesions. If a collision was detected at this stage, the colliding arc was simulated on the treatment unit as a further verification of model accuracy. A total of 88 patients have been tested using the collision detection application so far. The time required to run the automated collision test, from opening the software to receiving the results, was tracked using NLog logging software. Data logs were parsed for each clinical plan collision test to find the average runtime. A subset of 35 patients was also tested to compare the maximum allowable gantry trajectory using a 5 cm safety margin, to the clinically used gantry trajectory. Maximal clinical trajectories for noncoplanar treatments are currently guided by look‐up tables, which derive possible gantry‐couch combinations from worst‐case scenarios and apply them to the entire population. This solution is limiting on a patient‐specific basis as the majority of patients do not fall within this worst‐case scenario category.

## 3 RESULTS

### 3.1 Algorithm accuracy validation

**Figure 5:**
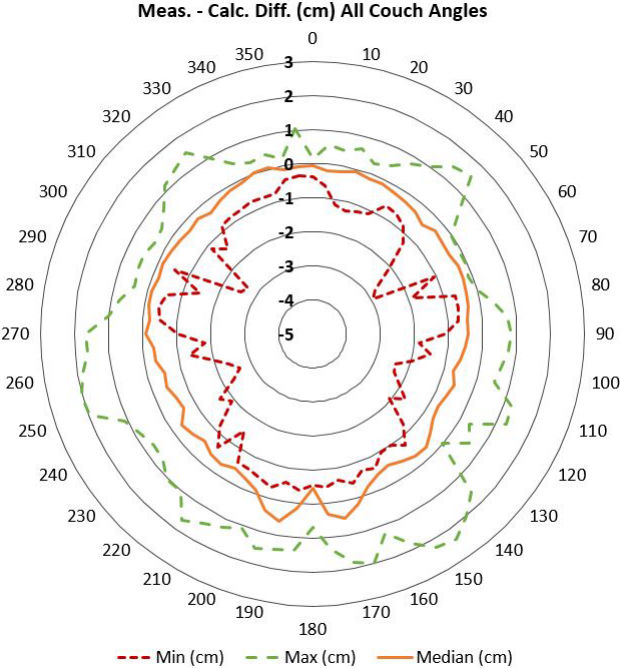
This radial plot shows the difference between the measured and calculated minimum distance between structures as a function of gantry angle. The minimum, maximum, and median differences between all allowable couch angles at that gantry angle are plotted. A negative shift implies overprediction of the minimum distance and the possibility of a collision being undetected.

The average difference between measured and calculated minimum distances for all gantry and couch angle combinations was −0.2 cm with a standard deviation of 0.7 cm. The largest overprediction was −2.9 cm and the largest underprediction was 2.4 cm. The minimum, maximum, and median difference for all allowable couch angles at each gantry angle can be seen in Fig. 5.

### 3.2 Clinical Validation

**Figure 6:**
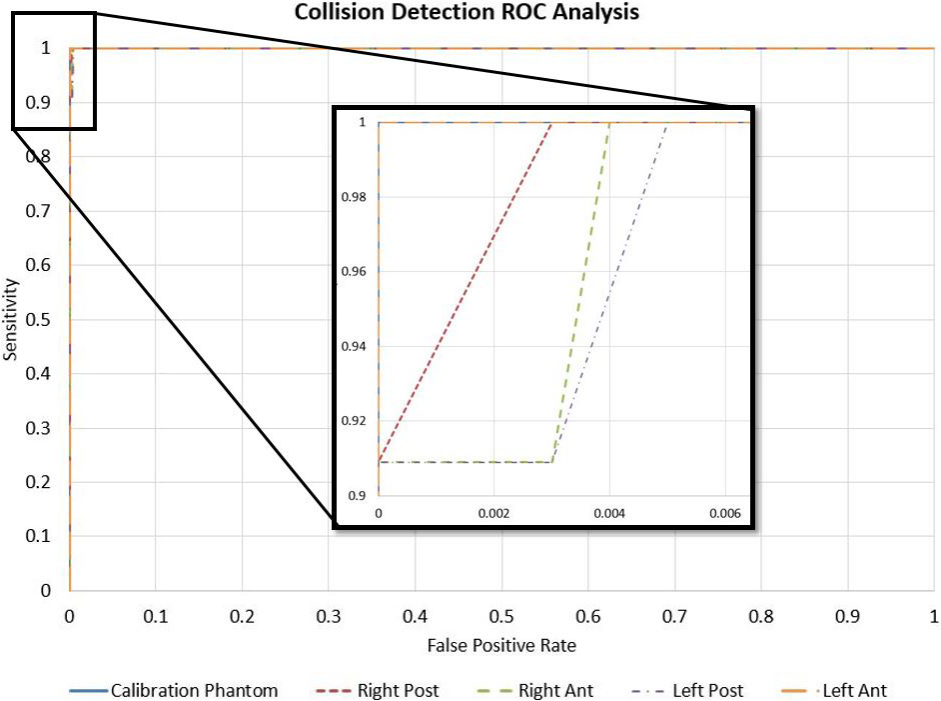
Receiver operating characteristic curves shown for both the calibration and anthropomorphic phantom validation testing with varying safety margins. 100% sensitivity is achieved for all cases with a 1.5 cm safety margin.

For the calibration phantom ROC curve, in Fig. 6, a safety margin of 1 cm resulted in 100% sensitivity and no false positives. The anthropomorphic phantom validation testing required a safety margin of 1.5 cm to achieve 100% sensitivity for all cases. The highest corresponding false positive rate for this margin was 0.9%. Table 2 shows ROC results with varying safety margins for the calibration and four test cases. At the clinical safety margin of 5 cm, algorithm accuracy dropped to a minimum of 94.2% which represents a 6% reduction in allowable gantry orientations. Larger safety margins reduce the number of false negative results with a subsequent increase in false positives. Appropriate margin choice is a trade‐off between preventing undetected collisions and reduction in accuracy.

**Table 2:** Receiver operating characteristic results for the calibration phantom and four different anthropomorphic phantom test cases with three different safety margins used. Accuracy is given by the sum of true positive (TP) and true negative (TN) results divided by the total for all results. Negative predictive value (NPV) = TN/(TN + FN) achieves unity for no false negative (FN) results.

	No safety margin			3 cm safety margin			5 cm safety margin		
	TP|TN|FP|FN	Accuracy (%)	NPV (%)	TP|TN|FP|FN	Accuracy (%)	NPV (%)	TP|TN|FP|FN	Accuracy (%)	NPV (%)
Calibration	45|709|0|17	97.8	97.7	62|709|0|0	100	100	62|697|12|0	98.4	100
Right post	7|648|0|4	99.4	99.4	11|638|10|0	98.5	100	11|629|19|0	97.1	100
Right ant	3|670|0|8	98.8	98.8	11|661|9|0	98.7	100	11|638|32|0	95.3	100
Left post	6|650|0|5	99.2	99.2	11|638|12|0	98.2	100	11|629|21|0	96.8	100
Left ant	4|683|0|7	99.0	99.0	11|666|17|0	97.6	100	11|643|40|0	94.2	100

### 3.3 Clinical implementation

**Figure 7:**
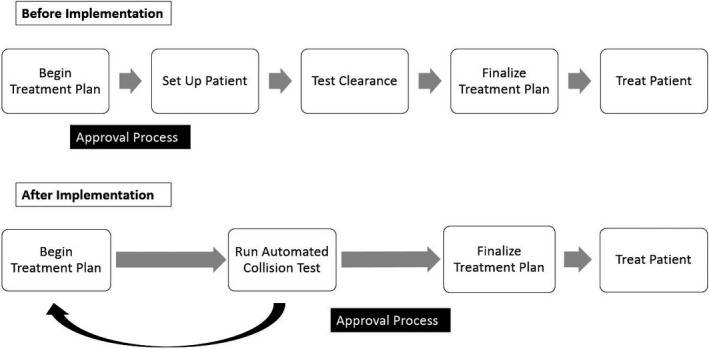
Change in clinical collision mitigation workflow from before and after implementing the collision detection application. Running the application takes an average of 2:58 s per plan.

During a 7‐month period, a total of 88 SRS patients totaling 107 SRS patient plans were tested using the collision detection application. The application was able to correctly detect and prevent one SRS collision during this period. In addition, no collisions occurred that were unpredicted by the application. Before implementation of the application, as shown in Fig. 7, this collision would have been detected after the clinical plan was approved and the patient was set up ready for treatment, resulting in a long re‐approval process and inefficiencies and redundancies in the process. With the application, clinically implemented collisions are now detected before the plan approval process, when changes to the clinical plan can be easily made, and with limited addition to planning time. On average, the total process from opening the patient, registering additional contours, and performing the collision check takes 2 min and 58 s per plan. Collision check software does not prevent navigation or use of the treatment planning system while in use. Substantial runtime improvements are achievable with upgraded hardware. Switching to Eclipse 15.5 with updated hardware resulted in the algorithm running 2.8 times faster on average for a test set of five patient plans. Runtime was reduced from 05:17 to 01:54 s.

The majority of SRS patient plans were found to be highly conservative with gantry trajectory. A study of 35 patients, with 46 corresponding plans, found that on average each arc could be increased by 76°, omitting arcs that were already a 360 degree trajectory.

## 4 DISCUSSION

This study improves on the accuracy of previous collision detection studies^4^, ^5^, ^9^ through the use of a detailed CAD gantry model and OBB Tree algorithm. Efficient manipulation of large triangle mesh structures is achieved by fast collision methods such as OBBs and bounding tree hierarchies. The collision detection application's distance accuracy was shown to be consistently within 3 cm of the measured true value. These results validated the use of a clinical safety margin of 5 cm as more than sufficient to account for any uncertainties in application accuracy. Sensitivity of the application to uncertainties was shown to be minimal using ROC analysis, as these uncertainties have a limited effect on the application's ability to predict actual collision events. Through ROC curve analysis, a 1.5 cm safety margin was shown to be sufficient in preventing any test case collisions from being undetected. The clinically used margin of 5 cm reduces the accuracy by limiting the usable treatment space; however, in a clinical setting, it is highly preferable to never come within 5 cm of the patient or couch with the gantry.

Previous studies^6^, ^7^, ^10^ have implemented a 3D scanning technique to acquire relatively accurate whole body contours, at the expense of scanning time and additional equipment cost. Yu et al. did not include any time analysis for scan acquisition using a single handheld scanner, nor was there an estimate for the total time required for scanning and plan analysis. Padilla et al. estimates a total time of 10 min for an experienced user. This includes scanning with a single Kinect camera, reconstruction, and manually registering the scan using virtual lasers; however, algorithm accuracy is limited by the use of a simple cylindrical gantry model. Cardan et al. reduce the whole process to less than a minute with the additional expense of three separate Kinect cameras and a high‐performance PC to run the collision detection simulation. CT simulation‐based techniques are limited by the scan length but require little additional clinical resources. This is the first study to enhance CT simulation scan length with the ability to automatically register detailed couch and immobilization structures to the body contour as well as add various sized body additions modeled from an anthropomorphic phantom. Furthermore, additional body shape approximation is possible by extending the CT contours in either the superior or inferior patient direction.

Eclipse Scripting allows the application to be highly integrated with clinical workflow. All patient plan data are directly available such that export of plan or structure set data is not required. The application was a stand‐alone executable and was designed such that other applications can be run in the Eclipse TPS simultaneously, to ensure maximum clinical efficiency. The entire collision detection process consisting of opening the patient, adding additional contours, and checking all beams for collisions takes approximately 3 min for a typical noncoplanar VMAT three arc SRS plan. Furthermore, this application can be used to inform planning decisions such as viability of isocenter location, as well as maximum gantry range testing for specific couch angles. Current clinical plans were shown to be conservative with gantry arc trajectory, which can now be advanced further using the application. This would expand allowable control points available for dose optimization and possibly improve plan quality.

The collision detection application has been purposefully designed to be easily usable at other clinics using the Truebeam system. The initial setup requires contours of immobilization structures used at the clinic, which are stored within the software for subsequent use. The use of our application for SRS technique was emphasized in this work but it can be expanded to any other treatment techniques or sites, provided the plan contours are a sufficient approximation of patient setup.

## 5 CONCLUSION

This study has demonstrated the viability and efficiency of a new patient contour‐based collision detection application during the treatment planning process. The use of Eclipse Scripting enables maximal integration with the current clinical workflow without any additional required resources. The limitations of the application due to the incomplete contour data are solved through auto‐registration of necessary additional structures. This application allows clinics to shift collision mitigation strategy from a passive and reactive to a proactive approach. Potential collisions are now discovered and prevented at the treatment planning stage of the process before they can negatively impact clinical resource utilization. Application utility is also easily expanded to other treatment sites and techniques using Varian TrueBeam with minimal setup requirements.

## CONFLICTS OF INTEREST

The authors confirm there are no relevant conflicts of interest to disclose.
